# 1787. Association of Lifestyle Factors with Sepsis Incidence: A Population-Based Cohort Study in South Korea

**DOI:** 10.1093/ofid/ofad500.1616

**Published:** 2023-11-27

**Authors:** Eun Hwa Lee, Kyoung Hwa Lee, Kyung-Do Han, Young Goo Song, Sang Hoon

**Affiliations:** Yonsei University College of Medicine, Seoul, Seoul-t'ukpyolsi, Republic of Korea; Yonsei University College of Medicine, Seoul, Republic of Korea, Seoul, Seoul-t'ukpyolsi, Republic of Korea; Department of Statistics and Actuarial Science, Soongsil University, Seoul, Korea, Seoul, Seoul-t'ukpyolsi, Republic of Korea; Yonsei University College of Medicine, Seoul, Seoul-t'ukpyolsi, Republic of Korea; Yonsei University College of Medicine, Seoul, Republic of Korea, Seoul, Seoul-t'ukpyolsi, Republic of Korea

## Abstract

**Background:**

Sepsis, a critically-ill syndrome that can cause damage to multiple organs, is a leading cause of death worldwide with significant healthcare costs. However, there was no large-scale study to evaluate the association of daily modifiable lifestyles with the risk of sepsis, which will be very important that the occurrence of sepsis can be sufficiently prevented by individual volition and effort.

**Methods:**

A retrospective population-based cohort study was performed to assess the association between the incidence rate (IR) of sepsis and alcohol consumption, tobacco smoking, and physical activity. Clinical data from health checkup arranged by the Korean National Health Insurance System (NHIS) were obtained from individuals 20 years or older in 2009 (n=4,234,415). The final 3,837,953 patients were analyzed, excluding personnel with sepsis during the 1 year before and after the checkup and patients with missing data. The NHIS claim data of all patients in the cohort were followed up until December 31, 2019. The primary outcome was the occurrence of sepsis as defined by the ICD-10 codes (A02, A32, A40, A41, A42, B37, R57, and R65). Lifestyle factors were collected through health-related behavior questionnaires in the health checkup.

**Results:**

During a median 9.32 years of follow-up, 78,188 sepsis cases were identified. The multivariate model, which adjusted for age, socioeconomic status, body mass index, diabetes mellitus, hypertension, and dyslipidemia, revealed that smoking was independently associated with a higher risk of developing sepsis in both male (adjusted hazard ratio: 1.29, 95% confidence interval: 1.26-1.32, *P*< 0.0001) and female (1.48, 1.40-1.56, *P*< 0.0001). Heavy drinking was associated with a higher IR in female (1.33, 1.18-1.51. *P*< 0.0001), but a lower IR in male (0.95, 0.92-0.97, *P*< 0.0001). The individuals who engage in regular exercise have a lower risk of developing sepsis in both male (0.94, 0.92-0.96, *P*< 0.0001), and female (0.89, 0.86-0.91, *P*< 0.0001) compared to those who reported physical inactivity.

**Figure:**
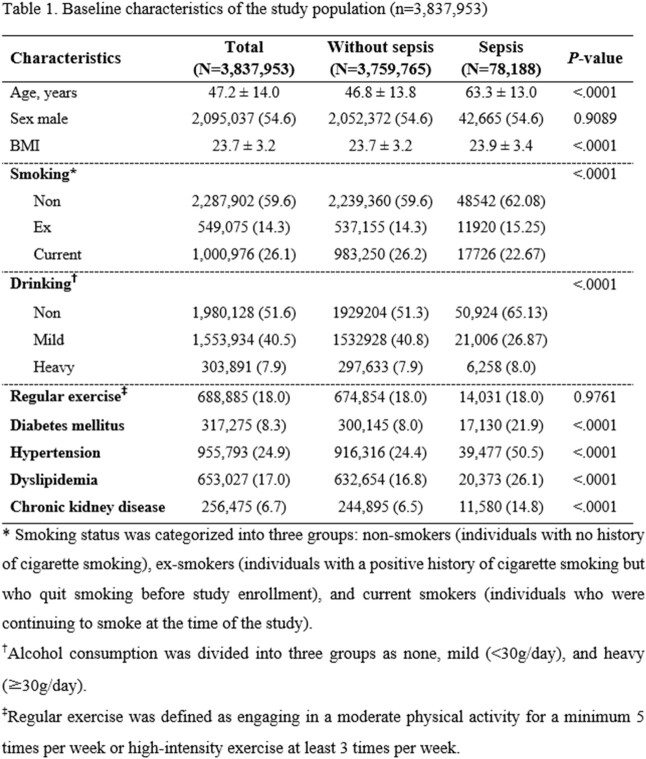

**Figure:**
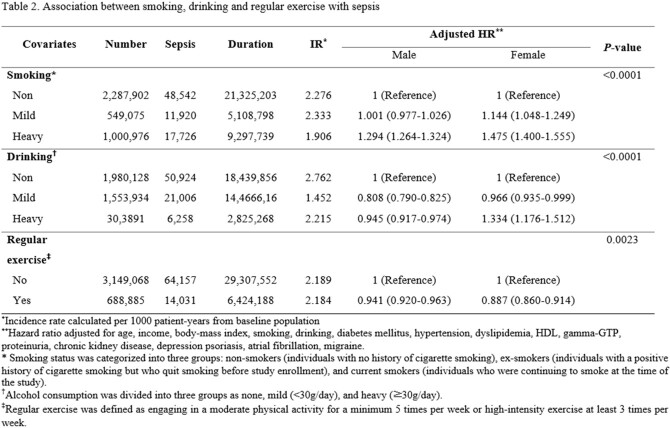

**Conclusion:**

This study suggests that lifestyle factors such as smoking, drinking, and regular exercise may play a role in the incidence of sepsis, and that modifying these factors may have potential benefits for preventing or reducing the incidence of sepsis.

**Disclosures:**

**All Authors**: No reported disclosures

